# *Cerrena unicolor* Laccases, Genes Expression and Regulation of Activity

**DOI:** 10.3390/biom11030468

**Published:** 2021-03-22

**Authors:** Anna Pawlik, Beata Ciołek, Justyna Sulej, Andrzej Mazur, Przemysław Grela, Magdalena Staszczak, Mateusz Niścior, Magdalena Jaszek, Anna Matuszewska, Grzegorz Janusz, Andrzej Paszczyński

**Affiliations:** 1 Department of Biochemistry and Biotechnology, Institute of Biological Sciences, Maria Curie-Skłodowska University, 20-033 Lublin, Poland; anna.pawlik@poczta.umcs.lublin.pl (A.P.); justyna.sulej@poczta.umcs.lublin.pl (J.S.); magdalena.staszczak@poczta.umcs.lublin.pl (M.S.); mateusz.niscior08@gmail.com (M.N.); magdalena.jaszek@poczta.umcs.lublin.pl (M.J.); anna.matuszewska@poczta.umcs.lublin.pl (A.M.); 2 Institute of Biological Sciences, Maria Curie-Skłodowska University, 20-033 Lublin, Poland; beata.rola07@gmail.com; 3 Department of Genetics and Microbiology, Institute of Biological Sciences, Maria Curie-Skłodowska University, 20-033 Lublin, Poland; mazur@hektor.umcs.lublin.pl; 4 Department of Molecular Biology, Institute of Biological Sciences, Maria Curie-Skłodowska University, 20-033 Lublin, Poland; przemek@hektor.umcs.lublin.pl; 5 Professor Emeritus, School of Food Science, University of Idaho and Washington State University, Moscow, ID 83843, USA; andrzej@uidaho.edu

**Keywords:** *Cerrena unicolor*, laccase, gene expression, proteomic, protease, PAGE, cyclic voltammetry

## Abstract

A white rot fungus *Cerrena unicolor* has been identified as an important source of laccase, unfortunately regulation of this enzyme genes expression is poorly understood. Using 1D and 2D PAGE and LC-MS/MS, laccase isoenzymes were investigated in the liquid filtrate of *C. unicolor* culture. The level of expression of laccase genes was measured using qPCR. The elevated concentrations of copper and manganese in the medium caused greatest change in genes expression and three laccase transcripts were significantly affected after culture temperature was decreased from 28 to 4 °C or increased to 40 °C. The small differences in the PAGE band intensities of individual laccase proteins were also observed, indicating that given compound affect particular laccase’s transcript. Analyses of laccase-specific activity, at all tested conditions, showed the increased activities as compared to the control, suggesting that enzyme is regulated at the post-translational stage. We observed that the aspartic protease purified from *C. unicolor*, significantly stimulate laccase activity. Moreover, electrochemical analysis of protease-treated laccase sample had 5 times higher redox peaks. The obtained results indicate that laccases released by *C. unicolor* are regulated at transcriptional, translational, and at the post-translational steps of gene expression helping fungus adapt to the environmental changes.

## 1. Introduction

*Cerrena unicolor* a white-rot basidiomycete, with parasitic or saprobic lifestyle, can attack several hardwood tree species, but also is proficient in decomposing dead softwoods’ logs. In order to decay a wooden tissue, fungus produce an array of hydrolytic and oxidative enzymes including: cellulases, xylanases, manganese peroxidase, lignin peroxidase, and the most extensively studied, laccase [1,2,3]. However, the coding potential of *C. unicolor* genome (https://mycocosm.jgi.doe.gov/Cerun2/Cerun2.home.html, accessed on 15 January 2021) suggests that the inventory of enzymes involved in wood degradation is far greater than the number of enzymes identified up to date. Our recent analysis of *C. unicolor* transcriptomes revealed about 300 transcripts coding for proteins putatively involved in sound wood degradation, of which one-fifth was differentially regulated depending on the type of wood substrate used [4]. Beside of lignolytic enzymes mentioned above, dye-decolorizing peroxidase, alcohol oxidase, and versatile peroxidase were identified, all are classified as lignin-metabolizing enzymes [4].

Attention has been drawn to biotechnological laccase production [1,2,3] and *C. unicolor* was identified as an organism capable of secretion of the high amounts of laccase in liquid culture [2,5]. Production of this enzyme was proven to be affected by aromatic acids [6], copper [7], cadmium [8], and temperature shock [9]. 

Over the past two decades, the number of reports describing applications of laccase in textiles industry [10], dye decolorization [11], wastes treatment [12], and in medicine [13] has noticeably increased. However, little is known about the physiology of *C. unicolor* and the role of laccase in its “lifestyle”. Analyses of genome and its annotation suggests existence of eight genes coding for laccase (Kyoto Encyclopedia of Genes and Genomes). However, in our previous work, up to 17 transcripts encoding putative laccases were found during *C. unicolor* growth in sawdust media [4]. Considering the importance of laccase [14] in wood degradation, it is vital to understand physiological role of *C. unicolor* laccases, particularly in lignin and xenobiotics degradation. Other possible biological functions of the enzyme, include involvement in fruit body formation [15] and response to environmental stress [16,17]. Recent findings suggest, that laccase primary role in living systems is protective function against adverse environmental factors including competitive or parasitic organisms and toxic compounds [17]. Little is also known about whether induction of laccase by metal ions and heat-shock occurs only at the stage of transcription (such regulation was described in *Trametes velutina* [18]) and which genes are involved. Fungal peroxidases and laccases have already been proven to be differentially regulated by the pH and temperature [19] or different media components [20]. Though, nutrient deficiency, copper, manganese, and aromatic compounds such as 2,5-xylidine and ferulic acid increase individual laccase gene transcripts [20,21]. Staszczak and Jarosz-Wilkolazka [22] proved that inhibition of proteasomal activity in *Trametes versicolor* may stimulate laccase synthesis by cadmium ions. In this paper, we compared laccase genes expression with protein analysis and activity in *C. unicolor* at different growth conditions. We postulated earlier that laccase activity in this fungus may be regulated by proteases [23,24], therefore, purified endogenous aspartic-protease was incubated with *C. unicolor* laccases and the resulting digest was analyzed by biochemical and electrochemical methods. Results suggest that laccase synthesis by *C. unicolor* is regulated at transcriptional, translational, and post-transcriptional stages of the protein synthesis.

## 2. Materials and Methods

### 2.1. Growth Conditions

The strain FCL139 of *Cerrena unicolor* was obtained from the culture collection of the Regensburg University and maintained on 2% (*w*/*v*) malt agar slants. A piece of 0.5 cm diameter of agar hyphae per conical flask were used to inoculate Lindenberg and Holm medium [25] and the culture was grown in non-agitated conditions for 7 days at 28 °C. Mycelial mats were collected and homogenized in a disperser (IKA, Poland) and homogenate of mycelial suspension (2.5% *v*/*v*) was used as an inoculum for all studies. The fungus was grown in an orbital rotary shaker (INFORS, Bottmingen-Basel, Switzerland) at 200 rpm for 8 days at 28 °C in 100 mL Erlenmeyer flasks containing 40 mL of the Lindeberg and Holm medium. Each of the culture variants was performed in triplicates.

All stock solutions of tested compounds were prepared in water (CdCl_2_, MnCl_2_, and CuSO_4_) or in ethanol (veratric and ferulic acids) and sterilized by filtration through Sterivex-GS filters (pore size, 0.22 μm; Millipore Corp.). Individual solutions were added to the fungal cultures on the sixth day of growth, and the growth was continued for the next 2 days. Final concentrations in the media were 1.0 mM for veratric and ferulic acids, and 10 μM for CdCl_2_, MnCl_2_, and CuSO_4_. The final concentration of ethanol in the growth medium was always less than 0.5%, and an equivalent amount of ethanol was added to control cultures without the aromatic acid. The influence of low and high temperatures was also evaluated on the sixth day of growth, and the cultures were subjected to 4 or 40 °C for 2 h before analyses were performed [26]. The culture filtrates were collected using Miracloth (Sigma Chemical Co., St. Louis, MO, USA) and centrifuged (at 6000× *g*, 4 °C) for 20 min in order to remove hyphal fragments. Resulting liquid was used for proteomic and the other analyses.

### 2.2. Laccase Activity and Protein Assay

Laccase activity was measured in triplicates, using an Infinite 200 Pro microplate reader (Tecan, Männedorf, Switzerland) in three separate biological replications with 25 µM syringaldazine (4-hydroxy, 3,5-dimetoxybenzaldehyde) (Aldrich, St. Louis, MO, USA) as a substrate, suspended in 0.1 mM, citrate-phosphate buffer pH 5.3 [27]. The formation of oxidation product was recorded at 525 nm at 20 °C. Laccase activity was expressed as nkat/L. Protein concentration was determined using Coomassie Brilliant Blue G-250, dye-binding method [28].

Kinetics parameters (*K*_m_, *V*_max_) and electrochemical experiments for digested laccase were determined as described earlier [23].

### 2.3. Separation of Protein Samples by Native PAGE and SDS-PAGE

In order to separate and visualize the laccase isozymes in the culture native, 10% PAGE was used. The electrophoresis was conducted at 4 °C and 145 V. A 10-µg aliquot of the individual protein sample was loaded per gel lane. The laccase activity was visualized with 0.01% guaiacol (Sigma Chemical Co., St. Louis, MO, USA) in 0.1 M citrate-phosphate buffer (pH 5.3), at 20 °C. Simultaneously, 10% sodium dodecyl sulphate-polyacrylamide gel electrophoresis (SDS-PAGE) was performed as described by Laemmli [29]. After separation, proteins were visualized by silver [30] or Coomassie Brilliant Blue G-250 staining. Prestained protein ladder (Fermentas, Glen Burnie, MA, USA) was used as a molecular weight marker. A G:Box (Syngene, Frederick, MD, USA) gel documentation system was used for gel imaging. 

### 2.4. Peptide Sequencing by LC-MS/MS

Secretome analysis was performed after 8 days of growth. The culture filtrate was concentrated 10 times using 3 kDa ultrafiltration membrane of Vivaspin 20 (Millipore, Darmstadt, Germany) and then, after measuring protein concentration, volume containing 100 ug of protein was lyophilized in a FreeZone 12, Freeze Dryer (Labconco, Kansas City, MO, USA). The individual dry sample containing 100 µg of protein was suspended in 100 µL of 100 mM ammonium bicarbonate and reduced with 5 mM tris[2 -carboxyethyl]phosphine-HCl (TCEP) for 1 h at 60 °C, blocked with 10 mM methyl methanethiosulfonate (MMTS) for 10 min, and digested overnight with 10 ng/uL mass spectrometry-grade trypsin. Resulting peptides mixture was analyzed using UPLC (NanoACQUITY, Waters, Milford, MA, USA) coupled with an Orbitrap Velos mass spectrometer. The resulted output list of precursor and product ions was compared with protein database of *C. unicolor* (12,978 sequences: 5,371,935 residues) using the local MASCOT server (version 2.4.1). The *C. unicolor* v1.1 (Cerun2) genome assembly was downloaded from the U.S. Department of Energy, Joint Genome Institute (DOE JGI, http://jgi.doe.gov, accessed on 16 January 2016) and used as the reference for the peptide sequences mapping. The MS/MS analysis was performed in the Environmental Laboratory of Mass Spectrometry, Institute of Biochemistry and Biophysics of Polish Academy of Sciences in Warsaw, Poland. The protein score cut-off was set for 150.

### 2.5. RNA Isolation and RT-PCR

Six-day-old cultures of *C. unicolor* were treated as described earlier, and RNA was extracted from the mycelium after 24 h of induction. A control experiment without additions was also performed. Each sample in 2.0 mL Lysing Matrix A tubes (MP Biomedicals, Solon, USA) was homogenized in a FastPrep-24 homogenizer (MP Biomedicals, Solon, OH, USA) in 0.5 mL of lysis buffer (Qiagen, Hilden, Germany) for 2 × 20 s at the 5 m/s rotor speed. After centrifugation (10,000× *g*, 4 °C, 8 min), a supernatant was used for the automated total RNA purification using an RNA Plus Mini Kit (Qiagen, Hilden, Germany) and a QIAcube robotic workstation (Qiagen, Hilden, Germany) according to the manufacturer’s protocol, which include a genomic DNA digestion step with RNase-Free DNase Set (Qiagen, Hilden, Germany). The obtained RNA was analyzed using a Qubit 2.0 fluorimeter and a Qubit RNA HS Assay Kit (Thermo Fisher Scientific, Waltham, MA, USA), as well as an Agilent 2100 Bioanalyzer and RNA 6000 Nano Kit (Agilent Technologies, Santa Clara, CA, USA). Single-stranded complementary DNA was synthesized from 1 µg of total RNA using a SuperScript^®^ VILO cDNA Synthesis Kit (Invitrogen, Carlsbad, CA, USA), according to the manufacturer’s protocol.

A StepOne™ Real-Time PCR System (Applied Biosystems, Foster City, CA, USA) was used for the quantitative analysis of mRNA expression. The qPCR reaction was carried out with 1 μL of cDNA (1:20 diluted) in a 25 µL reaction volume using the SYBR Green JumpStart Taq ReadyMix (Sigma, St. Louis, MO, USA). For amplification of the laccase transcripts, a set of specific primers (Table S1) was used. Primes were designed with Lasergene v.8.0.2 software (DNASTAR Inc., Madison, WI, USA). The standard curves of serial 1:10 dilutions of DNA templates (Eamp = 10^(-1/slope)^) [31] were used to calculated PCR efficiencies. Quantification of the mRNA was performed using the comparative Ct method (DDCt) with a reference gene coding for *β*-actin. Primers for the housekeeping *β*-actin gene were selected (Table S1) based on the available GenBank sequence. Amplification was carried out as follows: 40 cycles of 94 °C for 15 s and 60 °C for 1 min with an initial denaturation cycle of 94 °C for 2 min. All qPCRs were performed in a MicroAmp optical 96-well reaction plate with PE optical caps (Applied Biosystems, Foster City, CA, USA). For each experiment, a no-template reaction (NTC) was included as a negative control. All reactions were performed in triplicate.

The promoter regions sequences of *C. unicolor* 303 laccases were retrieved from the 1000 Fungal Genomes Project website (genome.jgi.doe.gov/programs/fungi/index.jsf, accessed on 16 June 2015). Nucleic acid sequences were analyzed using Lasergene v.8.0 software (DNASTAR, Inc., Madison, WI, USA). Database searches at the National Centre for Biotechnology Information (Bethesda, MD, USA) and the European Bioinformatics Institute (Hinxton, UK) were performed with the BLAST and FASTA.

### 2.6. 2-D gel Electrophoresis

Proteins from mycelium free culture fluid was concentrated by two ultrafiltration steps, first using Vivaspin 20, MWCO 3 kDa (PES; Millipore, Germany) and then Amicon Ultra-0.5 3K (Millipore, Darmstadt, Germany).

Each 5 µL aliquot of a concentrated protein sample was mixed with 125 µL of rehydration buffer containing 8 M urea, 2% CHAPS, 50 mM DTT, 0.2% (*w*/*v*) Bio-Lyte ampholyte solution (Bio-Rad, Hercules, CA, USA), and Bromophenol Blue (trace). The mixture was incubated for 30 min at 4 °C and protein sample in rehydration buffer (65 µg protein in 125 µL) was loaded onto a 7-cm IPG Ready Strip pH 3–6 (Bio-Rad, Hercules, CA, USA), covered with 2 mL of mineral oil, and then actively rehydrated for 12 h at 50 V and 20 °C, in a Protein IEF Cell (Bio-Rad, Hercules, CA, USA).

The IEF was carried out at 20 °C with a 50 µA per IPG strip limit. The settings for IEF were 250 V for 20 min (linear ramping), 4000 V for 2 h (linear ramping), and finally 4000 V for 10,000 Vh (rapid ramping). Immediately following IEF, the IPG strip was equilibrated in 2 mL of equilibration buffer I (0.375 M Tris-HCl pH 8.8, 6 M urea, 2% SDS, 20% (*v*/*v*) glycerol, and 2% DTT) and then in 2 mL of equilibration buffer II (the same equilibration buffer containing 2.5% iodoacetamide instead of DTT) for 15 min, each step on a shaker. Iodoacetamide and DTT were dissolved in equilibration buffers about 15 min before use.

Prior to second dimension separation, the IPG strips were rinsed in SDS running buffer (25 mM Tris base, 192 mM glycine, 0.1% (*w*/*v*) SDS) and then in the same buffer containing 0.0015% Bromophenol Blue. The IPG strips were placed on 12% gels (acrylamide-bisacrylamide at a ratio of 29:1) and electrophoresed at 150 V for 2 h. As a molecular mass marker, the Thermo Scientific PageRuler Prestained Protein Ladder was used. After electrophoresis, the proteins were visualized by staining with Coomassie Brilliant Blue R-250 (0.05% Coomassie, 25% isopropanol, and 10% acetic acid) using microwave-assisted method according to [32]. Images of gels were taken with ChemiDoc^TM^ MP Imaging System (Bio-Rad, Hercules, CA, USA).

### 2.7. Purification of Endogenous Aspartic-Like Protease from the C. unicolor Culture Fluid

At the first stage of purification, the post-culture fluid was centrifuged at 10,000× *g* on a 6K15 (Sigma, Osterode am Harz, Germany) centrifuge for 15 min at 4 °C. Then, the supernatant was concentrated by ultrafiltration, using Pellicon 2 Mini holder with an Ultracel mini cartridge (cut-off 10 kDa) (Millipore, Bedford, MA, USA). The extracellular aspartic-like protease was purified in the two chromatographic steps using FPLC, EconoSystem (Bio-Rad, Richmond, VA). The concentrated liquid was applied on a DEAE-Sepharose (Fast Flow) (2.5 cm × 15 cm) pre-equilibrated with 50 mM acetate buffer pH 5.0 and elution of the bound proteins was conducted with a linear gradient (0–0.5 M NaCl) at flow rate of 1 mL/min. Zymographic and spectrophotometric determination of the protease and laccase activity in the obtained fractions allowed for identification of the aspartic-like protease activity in the wash fractions and selecting it for the further purification. In the second stage of purification, the same column pre-equilibrated with 50 mM Tris-HCl (pH-7.4) was used. After the elution with a step gradient (1 M NaCl), one protein peak was observed. The peak fractions were pulled and desalted using Sephadex G25 and then lyophilized on a FreeZone 18 system (Labconco, Kansas City, MO, USA).

### 2.8. Measurements of Protease Activity

The protease activity was assayed using 1% hemoglobin solution in 0.1 M citrate-phosphate buffer at 3.5 pH according to the Anson [33]. After incubation of 0.2 mL of sample for 60 min with 0.5 mL of substrate, the 2 mL 5% TCA was added. The precipitated undigested proteins were centrifuged out and peptide and amino acid residues remaining in solution were detected spectrophotometrically at 280 nm (1U of protease activity was equal to the amount of the enzyme able to 0.01 AU increase in 1 min/1 mg of protein). For the qualitative characterization of the appropriate protease inhibitors (pepstatin, E-64, phenanthroline, EDTA, PMSF) in the 0.1 M citrate-phosphate buffer at pH 3.5 and 5.4 or 100 mM Tris-HCl at pH 9.5 were used. After 30 min incubations with given inhibitor, the proteolytic activity of sample was measured. The protease influence on laccase proteins was determined also using 12% native PAGE separation. Electrophoretic separation of 20 µg of each sample deposited per lane was performed for 45 min at 145 V and 4 °C. Protease activity was visualized after the incubation of gels in 0.1 M citrate-phosphate buffer (pH 3.5), at 37 °C containing a 1% Coomassie Brilliant Blue (R250), Heussen and Dowdle [34]. Analysis of the gel scans was performed with a G:Box (Syngene, Frederick, MD, USA).

### 2.9. Laccase-Protease Experiments

The solution of the laccase (0.25 U/mg) in water was incubated in a protein ratio of 1:1 (*v*/*v*) with the solution of aspartic-like protease (0.15 U/mg) at 37 °C. In the protease-treated samples laccase activity was determined at 60 and 120 min of incubation as was described earlier [23], and resulting reaction mixtures were subjected to further kinetic and electrochemical analysis with appropriate controls.

## 3. Results

### 3.1. LC-MS/MS Analyses

The extracellular proteins of *C. unicolor* were analyzed by LC-MS/MS. Based on the presence of unique peptides, five laccase isoenzymes and one aspartic protease were identified (Table 1). The identified laccase enzymes had similar molecular weight but differed in pI, which ranged from 4.44 to 5.92 pH. The calculated MW of all identified laccases was ca. 55 kDa, except one (protein ID 418196), whose mass seemed to be rather low (23 kDa) as for fungal laccase. Although, detailed analysis of gene coding for this protein (*C. unicolor* 303 genome) revealed repetitive sequence of unidentified nucleotides, which suggest that molecular weight of this laccase is higher than initially predicted.

### 3.2. Analysis of Differentially Expressed Laccase Genes

The production of laccases proteins detected by LC-MS/MS in the *C. unicolor* culture filtrates were compared with qPCR analyses, which employed a set of specific primers targeting relevant mRNA transcripts (Table S1). The transcription level of individual genes was measured in cultures containing aromatic acids (ferulic or verartic), metal ions (cadmium, manganese, or copper) or after incubation at 4 and 40 °C. A classical approach was used comprising the delta–delta Ct method under a paired experimental design (treatment vs. control).

**Figure 1:**
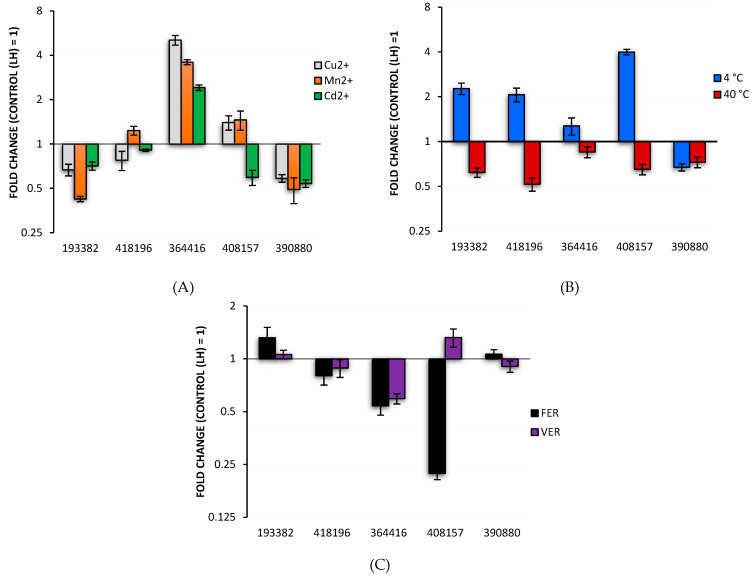
Effect of (**A**) Cu, Mn, and Cd ions, (**B**) temperature of 4 and 40 °C, and (**C**) ferulic (FER) and veratric (VER) acids on the expression of seven *C. unicolor* laccase genes. Error bars represent the standard deviations of the mean ± SD for three independent measurements (*n* = 3). LH – Lindeberg and Holm medium.

Only one gene coding for protein 364416 was found to be upregulated (5 folds) by all the three metal ions. The transcription of genes coding for laccases, 408157 and 418196 were slightly induced by copper and/or manganese ions. Temperatures affected laccase gene transcription in a less specific way (Figure 1b). The transcription of four genes coding for laccases was upregulated at 4 °C and downregulated at 40 °C. However, only transcript for laccase 390880 was repressed when fungus was grown in both temperatures. Ferulic acid upregulated the transcription of 193382 gene, whereas veratric acid stimulated only the expression of the 408157 laccase coding gene (Figure 1c). Summarizing, aromatic acids slightly downregulated the transcription of majority laccase coding genes. It seemed that gene coding for protein of 418196 gene was least influenced, whereas the expression of 364416 and 40157 genes were most frequently affected by investigated factors.

### 3.3. Laccase Enzymatic Activity and Electrophoretic Profiles

**Figure 2:**
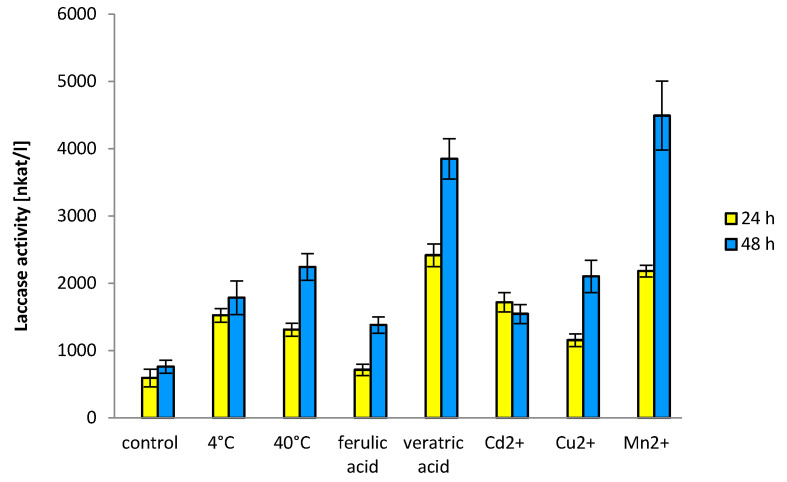
Effect of temperature, ferulic and veratric acids, Cu, Mn, and Cd ions on enzymatic activities of laccase after 24 and 48 h after induction of *C. unicolor*. Error bars represent the standard deviation of the mean ± SD for three independent measurements (n = 3).

The qPCR analyses have shown some small changes in laccase gene expression after 24 h, therefore, we also assayed laccase activity 48 h after induction. All tested variants resulted in higher activities of laccase after 48 h in the fungal cultures (Figure 2). The results showed that the addition of manganese or veratric acid stimulated laccase activities after 48 h, up to 9 and 7 times, respectively, as compared to the control. Only slight increase in laccase activity was observed after incubation of *C. unicolor* culture at 40 °C as compared to 4 °C.

**Figure 3:**
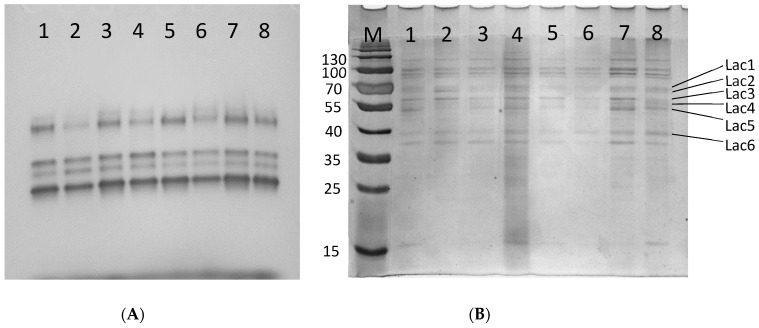
Laccase activity staining (**A**) and SDS-PAGE profile (**B**) of proteins found in the post-culture liquid of *C. unicolor*; 1, control (no induction); 2, 4 °C; 3, 40 °C; 4, ferulic acid; 5, veratric acid; 6, Cd; 7, Cu; 8, Mn; laccase bands are indicated.

In the visualized electropherograms, slight variations in the band intensities of individual laccases were noticed after culture incubation at 4 °C and addition of cadmium ions as well as in the presence of ferulic or veratric acids (Figure 3a), indicating that these compounds affected the individual laccase transcripts.

The differences in the intensities of the putative laccase bands were visible when extracellular proteins from *C. unicolor* were analyzed with SDS-PAGE (Figure 3b). In culture variants, 4 °C and veratric acid, a lower amount of protein band (denoted Lac5) was observed, whereas manganese was added to the *C. unicolor* culture the band corresponding to Lac3 was less intensive. Moreover, the intensity of the putative Lac2 band at 4 °C suggested its overproduction. In other culture variants, slight differences in the protein amounts can be observed, indicating that compounds tested, most likely affect the translations level of individual laccases.

### 3.4. 2-D Electrophoresis and LC-MS/MS

**Figure 4:**
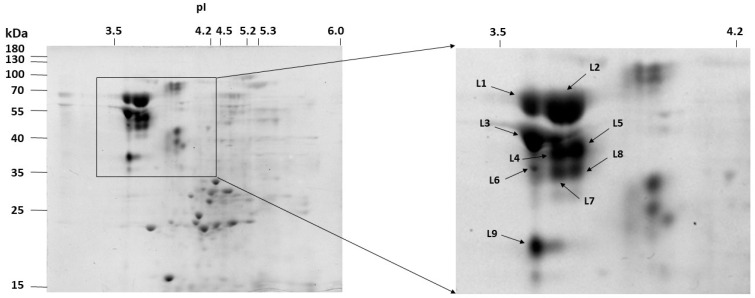
The 2D-gel separation of extracellular protein from the 8-day-old *C. unicolor culture*. Spots corresponding to the identified laccase enzymes are designated from L1 to L9.

The extracellular proteins from the uninduced, 8-day old *C. unicolor* culture was concentrated and analyzed using 2D-PAGE. Most proteins focused in a pH range of 3–6 and had molecular masses ranging from 15 to 130 kDa (Figure 4). Protein spots with activity toward guaiacol were cut and submitted to LC-MS/MS sequencing. Based on the presence of unique peptides corresponding to the gene sequences in the available *C. unicolor* genome, the same five laccase isoenzymes were identified (Table S2) as identified in the culture liquid before. Nine distinct spots (L1–L9) were observed with laccase activity and each consisted of several laccase proteins.

Despite predicted molecular weights ranging from 35 to 55 kDa, the laccase protein spots were observed between 35 and 70 kDa. The computed theoretical pI values of laccases ranged from 4.3 to 5.9, whereas the experimental pI values were found in a range from 3.5 to 4.0. It should be noted that both secretomic analysis performed for the total protein from the culture fluid and LC-MS/MS analysis of protein spots derived from 2D gels indicated existence of 23 kDa protein with putative laccase properties. However, the additional resequencing of the gene coding for this laccase was performed and the results proved that our strain (*C. unicolor* FCL139) genome contains complete sequence of laccase instead of ambiguous nucleotides sequence, as was reported earlier for this species. Therefore, the proteomic results of that spots should indicate protein of MW 55 kDa instead of 23 kDa.

### 3.5. Proteolytic Modifications of Laccase by C. unicolor Endogenous Protease

The steps described earlier for protease isolation allowed the separation of extracellular protein from *C. unicolor* culture with aspartic-like protease. The isolated protease showed high sensitivity to pepstatin (85.7% inhibition) which taken together with our LCMS/MS data suggest that it is aspartic-like protease (Table 1).

The purified laccase sample when mixed with purified protease showed increased activity to 146%, after 60 min incubation. The prolonged time (120 min) of incubation slightly elevated activity to 155%. The K_m_ of digested laccase decreased in time from 0.54 to 0.16 mM, after 120 min of incubation (Table 2). Similar phenomena were observed for V_max_ whose value dropped from 64.43 to 37.85 µM/min.

**Figure 5:**
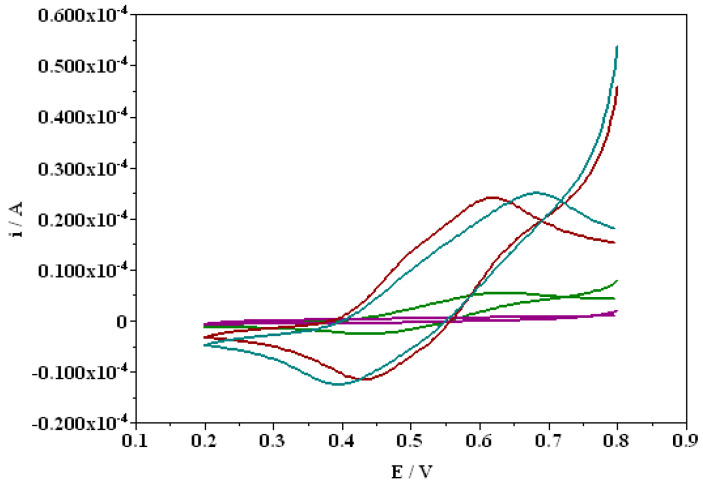
Electrochemical characterization of purified laccase isolated from *C. unicolor* after incubation with endogenous aspartic-like protease. Aspartic-like protease (violet line); laccase + ABTS (green line), after incubation time of 60 min (red line) and after 120 min (blue line).

Analysis of laccase redox potential was performed by cyclic voltamperometry (Figure 5). Slow-scan voltammograms were recorded in presence of 0.2 mM ABTS pH 5 with laccase and proteolytic enzyme-coated GCE (glassy carbon electrode). When laccase and protease were incubated for 60 min a well-shaped anodic peak (*E*_p,a_) with potential of 600 mV (vs Ag/AgCl) was observed. These peaks moved to 700 mV (vs Ag/AgCl) when enzymes were incubated for another 60 min (120 min in total). Similar difference was observed in case of cathodic peak (*E*_p,c_) at 400 mV (120 min incubation) and 450 mV (60 min incubation). Protease-treated laccase generated five times higher peaks than in the control experiment. The current measured in the presence of protease indicated a faster rate of the catalytic reaction.

## 4. Discussion

A number of reports indicate that *C. unicolor* culture is capable of production of high amount of extracellular laccase [7,35]. This enzyme plays an important role in lignolysis, and in other fungal physiologically processes, such as a response to harsh environmental conditions [9,36]. It was suggested that in *Polyporales* high number of laccases isozymes is the result of multiplication of a single ancestral gene and their further specialization [37]. Considering multifunctional laccase nature, it would be interesting to assign the expression of specific genes of individual laccases to their different physiological functions. In this work, the liquid medium of Lindeberg and Holm was chosen for *C. unicolor* growth, as it had already been successfully used earlier for the production of high amounts of laccase [7]. Surprisingly, only five laccases were detected in the culture medium, as demonstrated by LC-MS/MS analysis. Consequently, the genes encoding the identified laccases were further subjected to expression analysis at different temperature and the presence of metal ions and aromatic acids. It has already been shown that laccase synthesis may be influenced by high and low temperature [9,38]. As this fungus lives in the Northern hemisphere, where seasonal temperature fluctuates, low (4 °C) and high (40 °C) temperatures were chosen as extreme physical conditions. The obtained results demonstrated that laccase production is influenced by changing temperature in the fungus growth environment. Copper and cadmium ions have already been suggested to stimulate laccase production in *C. unicolor* cultures [7,8], which was also confirmed during this study. Different lignin derivatives were proposed to influence laccase synthesis in the genus *Cerrena* [6] and our research confirmed this phenomenon. Taking into consideration that laccase is lignin-degrading enzyme, it is surprising that not all laccase genes are upregulated by aromatic acids, but it seems correlated with our previous results when not all genes were influenced by different sawdust in the medium [4]. Our results support the concept that the laccase expression is the response to different environmental factors, occurs at both transcriptional and translational levels and enzyme is also regulated at post-translational stage. Since laccase synthesis is triggered by different chemical and physical factors, it is reasonable to expect that the promoter regions of respective genes containing various regulatory elements are responsible for regulation of expression. Although the genomic sequence of the *C. unicolor* FCL139 strain is not available, we searched the promoter regions of the genome of *C. unicolor* 303 to identify putative regulatory elements (Table S3). It was suggested that Heat Shock Elements (HSE) present in the laccase promoter may be involved in the response to temperature changes in the environment [39,40]. Although laccase gene, 390880 has the highest number of six HSE in its promoter region, its transcription is hardly affected by temperature in *C. unicolor* FCL139. Similarly, the promoter region of the laccase 193382, as the only one among the examined, possesses a putative ACE1 element responsible for regulation by copper ions, while the expression of laccase 364416 without ACE1 was strongly upregulated by all the metal ions used. Further detailed analysis of *C. unicolor* laccase expression is needed to understand their genes regulation. There is a chance that its genes may be clustered with other genes coding for wood degrading enzymes and therefore expressed together. Moreover, still little is known about laccase expression beside promoter elements. It should be emphasized that no metal-responsive elements (MRE) were found in the *C. unicolor* laccase promoters, which is in contrast with the respective laccase gene 364416 induction observed in the presence of cadmium. In the *C. unicolor* FCL139, only one laccase promoter region was sequenced and analyzed [41]. Comparing the respective promoter regions in *C. unicolor* 303 and FCL139 strains, slight differences were noticed in the number of hypothetical ACE1 and metal regulatory elements (MRE). Considering the observed differences in the DNA sequence of the respective promoter regions between fungal strains, further studies of laccase genes transcriptional regulations are needed, which may explain their contribution to the level of laccase biosynthesis. Additionally, the complexity of the regulation of *Cerrena unicolor* laccase synthesis and secretion results from the fact that the process is presumably affected by endogenous proteases [23,24], which was proved in taxonomically related *Trametes versicolor* [42]. We observed that in the *C. unicolor* culture filtrate (free of mycelium) laccase isoenzymes number, Osinska-Jaroszuk et al. [43], was reduced from five to two when incubated both in room and 4 °C temperature for prolonged time (up to 30 days). The obtained results in the presented manuscript of laccase activity assays, electrophoretic profile of laccases, and LC-MS/MS analyses demonstrated differences in enzyme biosynthesis in response to the tested factors. However, comparing these data to the results of transcriptional analyses, some differences can be observed, suggesting further, e.g., translational regulation of laccase activities. Therefore, based on LC-MS/MS data, one endogenous aspartic-protease was purified and used to digest laccase. The obtained results confirmed our previous observations [23], it seems that in nature laccase, kinetic and electrochemical properties may undergo post-translational proteolytical processing and despite 2 h incubation time, laccase was not completely digested as it was in case of animal proteinase K [23]. Perhaps, it is the way that hyphae recycle amino acid from secreted proteins since wood is considered a poor nitrogen source. It should be noted that the *C. unicolor* cultures exposed to different visible light conditions show also strong induction of laccase and protease activities [24]. Therefore, it is possible that proteases secreted by *C. unicolor* not only play role in plant matter digestion but also are an important post-translational enzymes regulator/recycler.

## 5. Conclusions

Our previous results and findings in this paper confirm that *C. unicolor* laccase plays an important role in fungus adaptation to harsh environmental conditions, including presence of metal ions or xenobiotics and adaptation to adverse physical factors such as temperature or light. We postulate that the number of laccase genes facilitate the saprotrophic lifestyle of the fungus allowing it to decompose several kinds of wood and different isoenzymes might promote adaptation to changing chemical and physical growth conditions. Even if recent studies emphasize transcriptional regulation of laccase expression, still little is known about post-translational processing and secretion of this enzyme. Therefore, further studies of laccase post-translational processing and activation by native proteases is warranted.

## Figures and Tables

**Table 1:** Laccases identified in the secretome of *C. unicolor* FCL139 by means of liquid chromatography–mass spectrometry (LC-MS/MS).

Protein ID *C. unicolor* 303	Transcript ID *C. unicolor* FCL139	Score	Query Coverage (%)	MW (Da)	pI
Laccase					
193382	XLOC_008690	8959	52	55139.50	4.64
390880	XLOC_011744	6576	37	54248.29	5.92
418196	XLOC_000669	3806	25	23445.25	4.44
408157	XLOC_011551	720	9	55484.45	5.08
364416	XLOC_011286	206	13	54798.69	5.20
Aspartic protease					
404065	XLOC_010406	218	9.5	56095.00	4.66

**Table 2:** Laccase kinetic constants after protease treatment.

	Time 0 min	Time 60 min	Time 120 min
K_m_ (mM)	0.54	0.19	0.16
V_max_ (µM/min)	64.43	43.46	37.85

## Supplementary Materials

The following are available online at https://www.mdpi.com/2218-273X/11/3/468/s1, **Table S1:** Primer sequences targeting individual laccase-coding genes used in qPCR and the length of amplification products, **Table S2:** Laccases identified in the secretome of *C. unicolor* FCL139 by means of 2D electrophoresis and liquid chromatography–mass spectrometry (LC-MS/MS), **Table S3:** Putative regulatory elements in the promoter regions of *Cerrena unicolor* 303 laccase genes.

## References

[1] Belova O.V., Lisov A.V., Vinokurova N.G., Kostenevich A.A., Sapunova L.I., Lobanok A.G., Leont’evskii A.A. (2014). Xylanase and cellulase of fungus Cerrena unicolor VKM F-3196: Production, properties, and applications for the saccharification of plant material. Prikl. Biokhim. Mikrobiol..

[2] Rogalski J., Dawidowicz A., Jozwik E., Leonowicz A. (1999). Immobilization of laccase from Cerrena unicolor on controlled porosity glass. J. Mol. Catal. B Enzym..

[3] Elisashvili V.I., Kvesitadze G.I. (1991). Laccase and Ligninase Activities of Cerrena-Unicolor. Bioconversion Plant Raw Mater. Biotechnol. Adv..

[4] Janusz G., Mazur A., Wielbo J., Koper P., Zebracki K., Pawlik A., Ciolek B., Paszczynski A., Kubik-Komar A. (2018). Comparative transcriptomic analysis of Cerrena unicolor revealed differential expression of genes engaged in degradation of various kinds of wood. Microbiol. Res..

[5] Fink-Boots M., Wilkolazka A., Malarczyk E., Leonowicz A. Changes in sets of laccase isoforms under stress conditions in Cerrena unicolor. Proceedings of the 7th International Conference on Biotechnology in the Pulp and Paper Industry.

[6] Yang J., Wang G., Ng T.B., Lin J., Ye X. (2015). Laccase Production and Differential Transcription of Laccase Genes in Cerrena sp. in Response to Metal Ions, Aromatic Compounds, and Nutrients. Front. Microbiol..

[7] Janusz G., Rogalski J., Szczodrak J. (2007). Increased production of laccase by Cerrena unicolor in submerged liquid cultures. World J. Microbiol. Biotechnol..

[8] Jarosz-Wilkolazka A., Graz M., Braha B., Menge S., Schlosser D., Krauss G.J. (2006). Species-specific Cd-stress response in the white rot basidiomycetes Abortiporus biennis and Cerrena unicolor. Biometals.

[9] Fink-Boots M.D., Jaszek M.B., Leonowicz A. (1997). Heat shock stimulation of laccase in Abortiporus biennis and Cerrena unicolor. Biol. Sci. Symp..

[10] Sojka-Ledakowicz J., Lichawska-Olczyk J., Ledakowicz S., Michniewicz A. (2007). Bio-scouring of linen fabrics with laccase complex from Cerrena unicolor. Fibres Text. East. Eur..

[11] Zhao M., Zhang B.B., Lu L., Zhao L.Y., Liang S.C. (2008). Purification and characterization of laccase from the white rot fungus Cerrena unicolor and its use in dye decolorization. J. Biotechnol..

[12] Mann J., Markham J.L., Peiris P., Spooner-Hart R.N., Holford P., Nair N.G. (2015). Use of olive mill wastewater as a suitable substrate for the production of laccase by Cerrena consors. Int. Biodeterior. Biodegrad..

[13] Matuszewska A., Karp M., Jaszek M., Janusz G., Osinska-Jaroszuk M., Sulej J., Stefaniuk D., Tomczak W., Giannopoulos K. (2016). Laccase purified from Cerrena unicolor exerts antitumor activity against leukemic cells. Oncol. Lett..

[14] Sharma K.K., Kuhad R.C. (2008). Laccase: Enzyme revisited and function redefined. Indian J. Microbiol..

[15] Leatham G.F., Stahmann M.A. (1981). Studies on the Laccase of Lentinus-Edodes—Specificity, Localization and Association with the Development of Fruiting Bodies. J. Gen. Microbiol..

[16] Missall T.A., Moran J.M., Corbett J.A., Lodge J.K. (2005). Distinct stress responses of two functional laccases in Cryptococcus neoformans are revealed in the absence of the thiol-specific antioxidant Tsa1. Eukaryot. Cell.

[17] Janusz G., Pawlik A., Swiderska-Burek U., Polak J., Sulej J., Jarosz-Wilkolazka A., Paszczynski A. (2020). Laccase Properties, Physiological Functions, and Evolution. Int. J. Mol. Sci..

[18] Yang Y., Wei F., Zhuo R., Fan F., Liu H., Zhang C., Ma L., Jiang M., Zhang X. (2013). Enhancing the laccase production and laccase gene expression in the white-rot fungus Trametes velutina 5930 with great potential for biotechnological applications by different metal ions and aromatic compounds. PLoS ONE.

[19] Fernandez-Fueyo E., Castanera R., Ruiz-Duenas F.J., Lopez-Lucendo M.F., Ramirez L., Pisabarro A.G., Martinez A.T. (2014). Ligninolytic peroxidase gene expression by Pleurotus ostreatus: Differential regulation in lignocellulose medium and effect of temperature and pH. Fungal Genet. Biol..

[20] Soden D.M., Dobson A.D.W. (2001). Differential regulation of laccase gene expression in Pleurotus sajor-caju. Microbiology.

[21] Swatek A., Staszczak M. (2020). Effect of Ferulic Acid, a Phenolic Inducer of Fungal Laccase, on 26S Proteasome Activities In Vitro. Int. J. Mol. Sci..

[22] Staszczak M., Jarosz-Wilkolazka A. (2005). Inhibition of the proteasome strongly affects cadmium stimulated laccase activity in Trametes versicolor. Biochimie.

[23] Janusz G., Jaszek M., Matuszewska A., Draczkowski P., Osinska-Jaroszuk M. (2015). Proteolytic modifications of laccase from Cerrena unicolor. J. Mol. Catal. B Enzym..

[24] Janusz G., Sulej J., Jaszek M., Osinska-Jaroszuk M. (2016). Effect of different wavelengths of light on laccase, cellobiose dehydrogenase, and proteases produced by Cerrena unicolor, Pycnoporus sanguineus and Phlebia lindtneri. Acta Biochim. Pol..

[25] Lindeberg G., Holm G. (1952). Occurrence of tyrosinase and laccase in fruit bodies and mycelia of some Hymenomycetes. Physiol. Plant..

[26] Jarosz-Wilkolazka A., Fink-Boots M., Malarczyk E., Leonowicz A. (1998). Formaldehyde as a proof and response to various kind of stress in some Basidiomycetes. Acta Biol. Hung..

[27] Leonowicz A., Grzywnowicz K. (1981). Quantitative estimation of laccase forms in some white-rot fungi using syringaldazine as a substrate. Enzym. Microb. Technol..

[28] Bradford M.M. (1976). A rapid and sensitive method for the quantitation of microgram quantities of protein utilizing the principle of protein-dye binding. Anal. Biochem..

[29] Laemmli U.K. (1970). Cleavage of structural proteins during the assembly of the head of bacteriophage T4. Nature.

[30] Walker J.M. (2009). The Protein Protocols Handbook.

[31] Pfaffl M.W. (2001). A new mathematical model for relative quantification in real-time RT-PCR. Nucleic Acids Res..

[32] Wong C., Sridhara S., Bardwell J.C., Jakob U. (2000). Heating greatly speeds Coomassie blue staining and destaining. Biotechniques.

[33] Anson M.L. (1938). The Estimation of Pepsin, Trypsin, Papain, and Cathepsin with Hemoglobin. J. Gen. Physiol..

[34] Heussen C., Dowdle E.B. (1980). Electrophoretic analysis of plasminogen activators in polyacrylamide gels containing sodium dodecyl sulfate and copolymerized substrates. Anal. Biochem..

[35] Songulashvili G., Spindler D., Jimenez-Tobon G.A., Jaspers C., Kerns G., Penninckx M.J. (2015). Production of a high level of laccase by submerged fermentation at 120-L scale of Cerrena unicolor C-139 grown on wheat bran. Comptes Rendus Biol..

[36] Filazzola M.T., Sannino F., Rao M.A., Gianfreda L. (1999). Effect of various pollutants and soil-like constituents on laccase from Cerrena unicolor. J. Environ. Qual..

[37] Savinova O.S., Moiseenko K.V., Vavilova E.A., Chulkin A.M., Fedorova T.V., Tyazhelova T.V., Vasina D.V. (2019). Evolutionary Relationships Between the Laccase Genes of Polyporales: Orthology-Based Classification of Laccase lsozymes and Functional Insight From Trametes hirsuta. Front. Microbiol..

[38] Hua S., Zhang B., Fu Y., Qi B., Li Y., Tian F. (2018). Enzymatic gene expression by Pleurotus tuoliensis (Bailinggu): Differential regulation under low temperature induction conditions. World J. Microbiol. Biotechnol..

[39] Piscitelli A., Giardina P., Lettera V., Pezzella C., Sannia G., Faraco V. (2011). Induction and transcriptional regulation of laccases in fungi. Curr. Genom..

[40] Janusz G., Kucharzyk K.H., Pawlik A., Staszczak M., Paszczynski A.J. (2013). Fungal laccase, manganese peroxidase and lignin peroxidase: Gene expression and regulation. Enzym. Microb. Technol..

[41] Janusz G., Mazur A., Checinsks A., Malek W., Rogalski J., Ohga S. (2012). Cloning and characterization of a laccase gene from biotechnologically important basidiomycete Cerrena unicolor. J. Fac. Agric. Kyushu Univ..

[42] Staszczak M., Zdunek E., Leonowicz A. (2000). Studies on the role of proteases in the white-rot fungus Trametes versicolor: Effect of PMSF and chloroquine on ligninolytic enzymes activity. J. Basic Microbiol..

[43] Osinska-Jaroszuk M., Jaszek M., Starosielec M., Sulej J., Matuszewska A., Janczarek M., Bancerz R., Wydrych J., Wiater A., Jarosz-Wilkolazka A. (2018). Bacterial exopolysaccharides as a modern biotechnological tool for modification of fungal laccase properties and metal ion binding. Bioprocess. Biosyst. Eng..

