# Supplementary material for: Cerrena unicolor Laccases, Genes Expression and Regulation of Activity
Source: Biomolecules. 2021 Mar 22;11(3):468. doi: 10.3390/biom11030468 (PMC8004220; doi:10.3390/biom11030468)
Supplement: Supplementary file 1 [file biomolecules-11-00468-s001.pdf]

Supplementary Materials

Cerrena Unicolor Laccases, Genes Expression and Regulation of Activity

Anna Pawlik [1], Beata Ciołek [2], Justyna Sulej [1], Andrzej Mazur [3], Przemysław Grela [4], Magdalena Staszczak [1], Mateusz Niścior [1], Magdalena Jaszek [1], Anna Matuszewska [1] Grzegorz Janusz [1,*] and Andrzej Paszczyński [5]

[1] Department of Biochemistry and Biotechnology, Institute of Biological Sciences, Maria Curie-Skłodowska University, 20-033 Lublin, Poland; anna.pawlik@poczta.umcs.lublin.pl (A.P.); justyna.sulej@poczta.umcs.lublin.pl (J.S.); magdalena.staszczak@poczta.umcs.lublin.pl (M.S.); mateusz.niscior08@gmail.com (M.N.); magdalena.jaszek@poczta.umcs.lublin.pl (M.J.); anna.matuszewska@poczta.umcs.lublin.pl; (A.M.)

[2] Institute of Biological Sciences, Maria Curie-Skłodowska University, 20-033 Lublin, Poland; beata.rola07@gmail.com

[3] Department of Genetics and Microbiology, Institute of Biological Sciences, Maria Curie-Skłodowska University, 20-033 Lublin, Poland; mazur@hektor.umcs.lublin.pl

[4] Department of Molecular Biology, Institute of Biological Sciences, Maria Curie-Skłodowska University, 20-033 Lublin, Poland; przemek@hektor.umcs.lublin.pl

[5] Professor Emeritus, School of Food Science, University of Idaho and Washington State University, Moscow, ID 83843, USA; andrzej@uidaho.edu

* Correspondence: gjanusz@poczta.umcs.lublin.pl: Tel.: +48-81-5375521

Table S1. Primer sequences targeting individual laccase-coding genes used in qPCR and the length of amplification products.

Gene	Primer sequence 5′-3′	Product size (bp)
β-actin	Fw CGAGGGCGACGTAGCAGAGC<br>Rev GATTTGGCCGGTCGTTGATTTGA	130
XLOC_000669	Fw CGCAGTGCTGGCCAAGATACTCC<br>Rev CGGGTTGTCGGTGGTGAAGCG	111
XLOC_008690	Fw CCCGGGCCATGGTTCCTTC<br>Rev GTAGGGTTCGCAGCTCTAGTCTCATTC	104
XLOC_011744	Fw CTAGGGCAAACGGGATACCAGAGA<br>Rev CCACCTTCGCCAATATTTTACATCC	124
XLOC_011551	Fw CGGTTCCGTCTGGTCAATATCG<br>Rev TAGAGTCAACAGTGTGGGGTTGGC	118
XLOC_011286	Fw CGGTGCCTCGTTCCTTGATCC<br>Rev TTCGACGACCTTGCCTAATTCGA	124

Table S2. Laccases identified in the secretome of *C. unicolor* FCL139 by means of 2D electrophoresis and LC-MS/MS.

Spot ID	Protein ID	Query coverage [%]	Score
L1	390880	25	4327
	193382	21	910
L2	390880	23	5410
	193382	14	616
L3	193382	31	7603
	418196	46	2540
	390880	25	1917
	364416	13	458
L4	390880	23	3751
	364416	13	700
	193382	16	699
L5	390880	25	5431
	193382	24	1181
	364416	13	688
	408157	12	532
L6	390880	25	3750
	193382	29	1629
	364416	10	256
L7	390880	25	5070
	193382	18	804
	364416	13	445
L8	390880	25	3957
	193382	11	491
	364416	14	340
L9	193382	27	5263
	390880	11	206

Proteins with less than 10% sequence coverage were excluded from the result

Table S3. Putative regulatory elements in the promoter regions of *Cerrena unicolor* 303 laccase genes.

	Putative regulatory elements [a]								
**promoter region/ laccase number**	**TATA**	**CAAT**	**NIT2**	**HSE**	**XRE**	**ACE1**	**Cre-A**	**STRE**	**MRE**
**scaffold_25:274118-276494 protein ID 357631**	-120	-509<br>-754<br>-894<br>-957<br>-1556			-1199				
**scaffold_52:16434-18852 protein ID 193382**		-159<br>-295<br>-386<br>-778<br>-932<br>-1186<br>-1682			-1768	-1598			
**scaffold_87:13089-15287 protein ID 364416**		-38<br>-406<br>-433<br>-613<br>-617<br>-739<br>-1465<br>-1585<br>-1688<br>-1726<br>-1758	-547		-1768				
**scaffold_87-23002-25174 protein ID 390832**	-76	-229<br>-583<br>-841<br>-890<br>-989<br>-1100<br>-1210<br>-1232<br>-1291			-1447			-1542	
**scaffold_96-28717-36266 protein ID 390880**	-75	-40<br>-645<br>-787<br>-1077	-1315	-50<br>-671<br>-783<br>-925<br>-944<br>-1254	-361<br>-1356		-1021<br>-1504		

**scaffold_9:516885-519056 protein ID 408157**	-369	-119<br>-238<br>-286<br>-481<br>-554<br>-683<br>-778<br>-998<br>-1560<br>-1863	
**scaffold_10:784280-786715 protein ID 418196**	-295	-331<br>-509<br>-883<br>-1524<br>-1547<br>-1590<br>-1598<br>-1993	-474<br>-755<br>-1974

[a] the relative position of a putative regulatory element is shown with respect to an individual laccase gene start codon
